# A Compact and High-Precision Three-Degree-of-Freedom Grating Encoder Based on a Quadrangular Frustum Pyramid Prism

**DOI:** 10.3390/s23084022

**Published:** 2023-04-15

**Authors:** Shengtong Wang, Baiqi Liao, Ningning Shi, Xinghui Li

**Affiliations:** 1 Tsinghua Shenzhen International Graduate School, Tsinghua University, Shenzhen 518055, China; wct21@mails.tsinghua.edu.cn (S.W.); lbq20@mails.tsinghua.edu.cn (B.L.);; 2 Tsinghua-Berkerley Shenzhen Institute, Tsinghua University, Shenzhen 518055, China

**Keywords:** displacement measurement, grating encoder, multi-degrees-of-freedom, compactness

## Abstract

A compact and high-precision three-degrees-of-freedom (DOF; X, Y, and Z directions) grating encoder based on the quadrangular frustum pyramid (QFP) prisms is proposed in this paper to solve the insufficient installation space problem of the reading head of the multi-DOF in high-precision displacement measurement applications. The encoder is based on the grating diffraction and interference principle, and a three-DOF measurement platform is built through the self-collimation function of the miniaturized QFP prism. The overall size of the reading head is 12.3 × 7.7 × 3 cm^3^ and has the potential for further miniaturization. The test results show that three-DOF measurements can be realized simultaneously in the range of X-250, Y-200, and Z-100 μm due to the limitations of the measurement grating size. The measurement accuracy of the main displacement is below 500 nm on average; the minimum and maximum errors are 0.0708% and 2.8422%, respectively. This design will help further popularize the research and applications of multi-DOF grating encoders in high-precision measurements.

## 1. Introduction

Precision displacement measurements play a remarkable role in national ultraprecision machining, national defense, and other fields [1,2,3]. At present, ultraprecision displacement measurements predominantly involve electrical and optical measurement methods. Electrical sensors typically include inductive, eddy current, and capacitive sensors, among which eddy current and capacitive displacement sensors are most commonly used for precision measurements. Eddy current is mainly aimed at the mm-level measurement range and can generally only provide micron accuracy [4,5,6,7]. Capacitive displacement sensors can achieve nanometer accuracy in a single degree-of-freedom (DOF). However, the measurement range is generally compressed to the level of hundreds of microns [8]. This compression is mainly due to the small linear range of the capacitance value [9]. Capacitive displacement sensors can also be used in three DOF, but only achieve micron-level accuracy [10]. A high-precision measurement of capacitive time grating is also currently observed, and the accuracy can reach submicron or even higher [11,12] in a stable electromagnetic environment. Current optical methods for precision displacement measurement include a laser interferometer and grating encoder, which both achieve nanometer or even higher accuracy in mm-level ranges with a complex system. Therefore, the two schemes have always complemented each other in the field of large-range precision measurements [13,14]. However, the laser interferometer is limited by the laser wavelength, which is the measurement standard [15,16]; therefore, in actual use, the wavelength is easily affected by the temperature and humidity in the environment, thus affecting accuracy [17,18,19]. Laser interferometers are generally used for measurements in a controlled environment in the laboratory, which can achieve high accuracy and stability [20,21,22]. The measurement standard of the grating encoder is the grating pitch [23,24]. The standard of this physical structure is substantially stable, and its measurement accuracy mainly depends on the accuracy and uniformity of the grating pitch [25,26,27,28]. In addition, absolute measurements are possible [29] with nanometer-level accuracy [30], and angle measurements can be realized under the appropriate optical path [31,32]. Therefore, a grating encoder is a widely used measurement method in actual industrial production and other applications.

However, in practical applications, the high-quality grating reading head will also increase the difficulty in controlling equipment motion due to the limited installation or operating space of production equipment; thus, high integration and stability of the grating encoder are required in most situations. Commercial products, such as Heidenhain, can only currently achieve multi-DOF by assembling together the sensor components [33]. Therefore, considering industrial applications, multi-DOF and integrated miniaturization are the largest obstacles to practical promotion.

Scholars have also conducted a series of research to achieve multi-DOF and compactness. Multi-DOF mostly comprises two to three DOF [34,35,36,37] and four to six DOF [32,38,39,40,41]. However, only a few scholars, mainly led by enterprises, such as the LIP6031D series products proposed by Heidenhain in July 2021, have researched miniaturization [42,43]. These products realize two-DOF for single reading head reads and assemble these sensors with low number DOF to form a sensor assembly with up to five DOF. The difficulty of miniaturization lies in the necessary batch collimation of diffracted light for the three-DOF grating encoder. Integrating this module and miniaturizing its volume is difficult. Scholars at home and abroad generally use prisms [40], convex lenses [44], or diffraction gratings [45] for batch collimation of diffracted light modules. Integrating the ordinary prism is complicated, and the convex lens needs focal length matching. This condition limits the miniaturization of the system and the low diffraction efficiency of the grating, which is not conducive to the detection of light intensity. Thus, the three solutions are not conducive to the development of miniaturization and integration.

This paper generally proposes a miniaturized three-DOF grating encoder based on quadrangular frustum pyramid (QFP) prisms to solve the urgent need for multi-DOF integration miniaturization and provides a reference for future six-DOF miniaturization. This encoder also addresses the difficulty of multi-DOF grating encoder application promotion and promotes research progress in multi-DOF miniaturization.

## 2. Principles and Method

**Figure 1:**
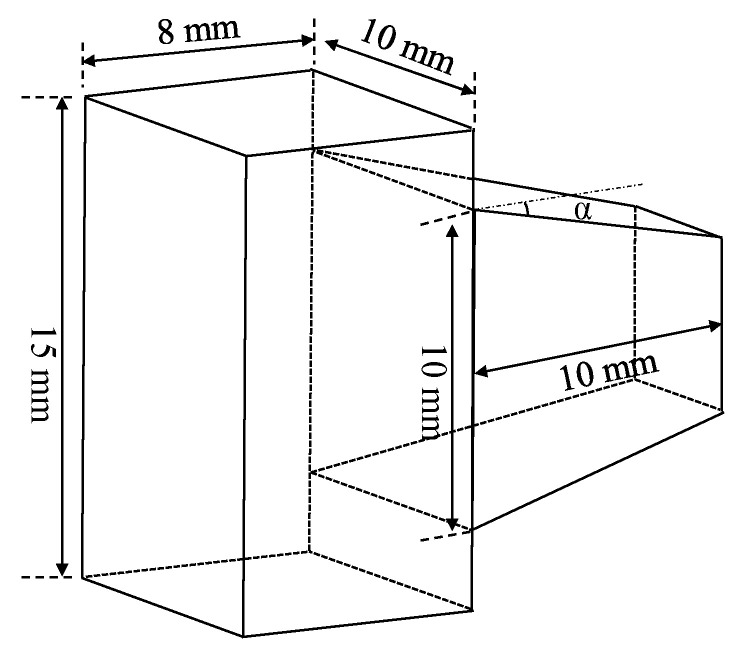
Schematic of the Quadrangular Frustum Pyramid Prism structure.

The main principle of the three-DOF compaction scheme proposed in this paper is to design the encoder using two QFP prisms. The schematic of the QFP prism is shown in Figure 1. The draft angle α of the collimated part is 12.924° when the collimation operation is performed corresponding to the diffraction angle of the 1-micron periodic grating. However, the experimental draft angle is approximately 12.9° because of the defects in the processing technology.

**Figure 2:**
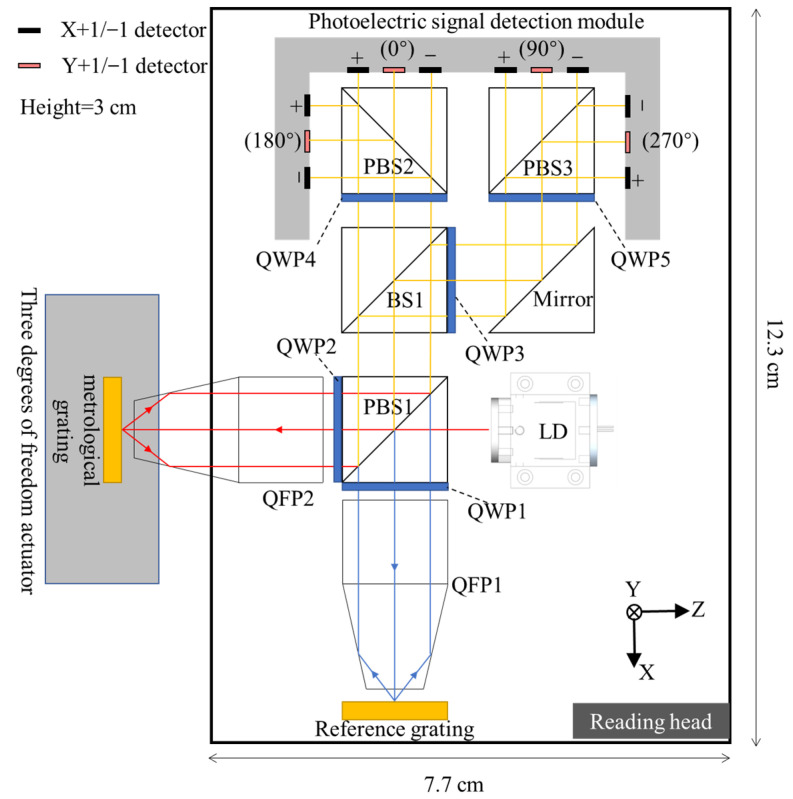
Schematic of the three-DOF reading head: (Beam Splitter (BS)).

This paper develops and designs a three-DOF encoder scheme using the QFP, and the schematic is shown in Figure 2. The main principle is as follows: the polarization state of the laser diode is adjusted when a laser with a wavelength of 660 nm is emitted from the laser diode (LD). Therefore, the energy of the P-light and S-light, which are separated by the polarizer beam splitter (PBS1), is the same. At this time, the S-light is first reflected, passed through a quarter-wave plate (QWP1), and then converted into circularly polarized light and irradiated on the reference grating. The four beams diffracted by the reference grating are X_+1_, X_−1_, Y_+1_, and Y_−1_. After collimation by QFP1, the four beams demonstrate parallel emission, pass through QWP1 again, and then transform into P-light to pass through PBS1. Similarly, the P-light from PBS1 follows the same rule. Finally, four parallel beams, namely X_+1_’, X_−1_’, Y_+1_’, and Y_−1_’, are obtained from the measurement grating, converted to S-light, and then reflected on PBS1 after passing through QWP2 again. At this time, two sets of four diffracted beams from various gratings enter the optical path subdivision module together under the performance of PBS1. These beams interfere with each other under the joint action of the QWP and PBS. Photoelectric signals are generated with phase information of 0°, 90°, 180°, and 270°. The four groups of signals can eliminate the influence of the energy fluctuation of the homodyne signal on the signal calculation and enhance the stability and accuracy of the signal. The displacement in the three directions of X, Y, and Z can finally be obtained in accordance with the signal calculation.

The phase change of the signal is used for measurement in three-DOF encoders. The Doppler frequency shift effect causes phase changes in the diffracted light direction when the grating measurement moves along the X and Y directions. The principles in the two directions are the same. Therefore, this paper takes the X direction as an example. When the grating measurement is displaced along the X direction, the phase of the diffracted lights $U_{S_{X\pm 1}}$ in the X direction changes, as shown in Equation (1), while the reference beams $U_{r_{X\pm 1}}$ remain unchanged.

(1) $$\left\{\begin{array}{l} U_{r_{X+1}}=U_{r_{X-1}}=U_{0}\\ U_{S_{X\pm 1}}=U_{0}e^{i}(\Omega_{X\pm 1}+\Phi ) \end{array}\right.,$$

where the phase changes are Ω and Φ, corresponding to the displacements in the X and Z directions, respectively.

(2) $$\left\{\begin{array}{l} \Omega_{X\pm 1}=\pm 2\pi \frac{\Delta x}{g}\\ \Phi =2\pi \frac{\Delta z(1+\cos\theta )}{\lambda } \end{array}\right.,$$

where ΔX and ΔZ are the displacements in the X and Z directions, respectively, g is the grating pitch, *θ* is the diffraction angle, and *λ* is the wavelength of the incident light.

After light synthesis,

(3) $$U_{X\pm 1}=Us_{X\pm 1}+Ur_{X\pm 1}.$$

The light intensity is

(4) $$I_{X\pm 1}=U_{X\pm 1}\cdot \bar{U_{X\pm 1}}=2U_{0}^{2}[1+cos\left(\Omega_{X\pm 1}+\Phi \right),$$

The light intensity information of the corresponding four phases is as follows:

(5) $$\left\{\begin{array}{l} I_{X\pm 1(0{}^{\circ})}=b_{1}+a_{1}\cos(\Omega_{\pm 1}+\Phi )\\ I_{X\pm 1(90{}^{\circ})}=b_{2}+a_{2}\cos(\Omega_{\pm 1}+\Phi +pi/2)\\ I_{X\pm 1(180{}^{\circ})}=b_{3}+a_{3}\cos(\Omega_{\pm 1}+\Phi +pi)\\ I_{X\pm 1(270{}^{\circ})}=b_{4}+a_{4}\cos(\Omega_{\pm 1}+\Phi +3\times pi/2) \end{array}\right.,$$

where *a*_1_–*a*_4_ is the amplitude of the ideal interference signal, and *b*_1_–*b*_4_ is the amplitude of the DC bias signal.

The influence of DC bias fluctuations can be removed, and the amplitude changes of the interference signal can be preserved.

(6) $$\left\{\begin{array}{l} S_{X+1}=\frac{I_{X+1}(0{}^{\circ})-I_{X+1}(180{}^{\circ})}{I_{X+1}(0{}^{\circ})+I_{X+1}(180{}^{\circ})},\\ S_{X+1}^{\prime }=\frac{I_{X+1}(90{}^{\circ})-I_{X+1}(270{}^{\circ})}{I_{X+1}(90{}^{\circ})+I_{X+1}(270{}^{\circ})},\\ S_{X-1}=\frac{I_{X-1}(0{}^{\circ})-I_{X-1}(180{}^{\circ})}{I_{X-1}(0{}^{\circ})+I_{X-1}(180{}^{\circ})},\\ S_{X-1}^{\prime }=\frac{I_{X-1}(90{}^{\circ})-I_{X-1}(270{}^{\circ})}{I_{X-1}(90{}^{\circ})+I_{X-1}(270{}^{\circ})}, \end{array}\right.$$

According to the trigonometric function, the calculation formula for the corresponding displacement can be obtained as follows:

(7) $$\left\{\begin{array}{l} \Delta X=\frac{g}{4\pi }\left\{\arctan\left(\frac{S_{X+1}}{S_{X+1}^{\prime }}\right)-\arctan\left(\frac{S_{X-1}}{S_{X-1}^{\prime }}\right)\right\},\\ \Delta Z=\frac{\lambda }{4\pi (1+\cos\theta )}\left\{\arctan\left(\frac{S_{X+1}}{S_{X+1}^{\prime }}\right)+\arctan\left(\frac{S_{X-1}}{S_{X-1}^{\prime }}\right)\right\}, \end{array}\right.$$

The calculation method in the Y direction is the same as that in the X direction and will not be repeated herein.

In actual data processing, direct data processing according to formulas (5)–(7) cannot obtain accurate displacement results because the actual incremental signal is not an ideal cosine signal. First, the results are affected by noise interference. Second, the optical component errors (e.g., the splitting ratio of the beam splitting prism is not ideal at 1:1), system installation errors (e.g., adjusting the angle between the fast axis of the wave plate and the X-axis direction to the optimum 45° is difficult), and errors in photoelectric conversion also contribute to the aforementioned phenomenon. The signal can be expressed as follows:

(8) $$S_{X+1}=\frac{I_{{X+1(180}^{o})}-I_{{X+1(0}^{o})}}{I_{{X+1(180}^{o})}+I_{{X+1(0}^{o})}}\approx A_{1}\sin(\Omega +\Phi )+B_{1},$$

(9) $${S_{X+1}}^{\prime }=\frac{I_{{X+1(90}^{o})}-I_{{X+1(270}^{o})}}{I_{{X+1(90}^{o})}+I_{{X+1(270}^{o})}}\approx A_{3}\cos(\Omega +\varphi -\Phi )+B_{3},$$

(10) $$S_{X-1}=\frac{I_{X-{1(180}^{o})}-I_{X-{1(0}^{o})}}{I_{X-{1(180}^{o})}+I_{X-{1(0}^{o})}}\approx A_{2}\sin(-\Omega +\Phi )+B_{2},$$

(11) $${S_{X-1}}^{\prime }=\frac{I_{X-{1(90}^{o})}-I_{X-{1(270}^{o})}}{I_{X-{1(90}^{o})}+I_{X-{1(270}^{o})}}\approx A_{4}\cos(-\Omega +\Phi -\sigma )+B_{4},$$

where *A*_1_–*A*_4_ is the fluctuation amplitude of the interference signal, σ is the phase deviation from the ideal signal, and *B*_1_–*B*_4_ is the maintained DC component.

Therefore, the signal processing process must be performed to minimize the relative error of the calculated results to the true value. First, the high-frequency noise signal will be removed by the low-pass filtering method without changing the phase. Second, amplitude regularization will generally correct the maximum absolute value of the signal to maintain consistency. Furthermore, the phase compensation can adjust the phase information of each signal according to the ideal phase set. Finally, arctangent counting will provide a displacement result based on the grating pitch.

## 3. Experiments and Discussion

**Figure 3:**
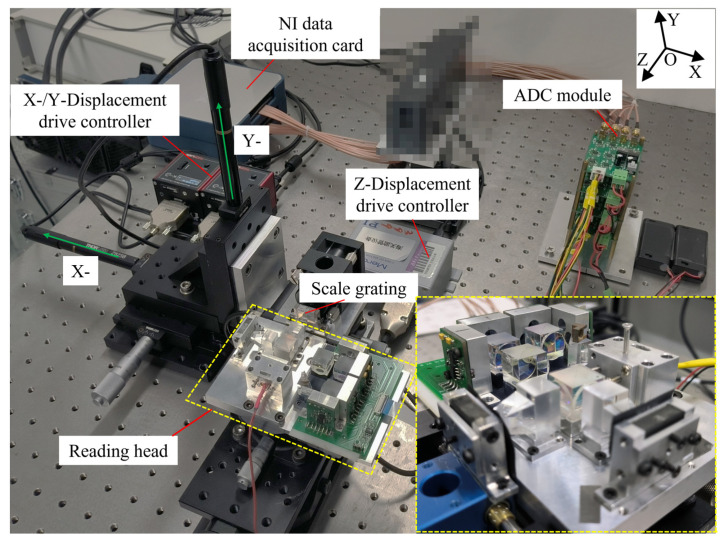
Three-DOF grating encoder test platform.

This paper designed the experiments in three DOF based on the above principles, and the object pictures of the test bench are shown in Figure 3. The pixelated part is a fixed device that has nothing to do with this paper.

The test platform comprises the following three main parts: data acquisition, displacement, and reading head. The data acquisition part mainly includes an NI acquisition card, an ADC module, and a computer. The displacement part is the Advanced Positioning Technology (APT) drive controller of THORLABS and the corresponding displacement drive (X and Y directions). The Z direction is controlled and displaced by the micron displacement stage of the Physik Instrumente (PI). The main parameters are shown in Table 1. The lower right corner of Figure 3 shows an enlarged picture of the reading head, which was built in accordance with the schematic of Figure 2.

**Figure 4:**
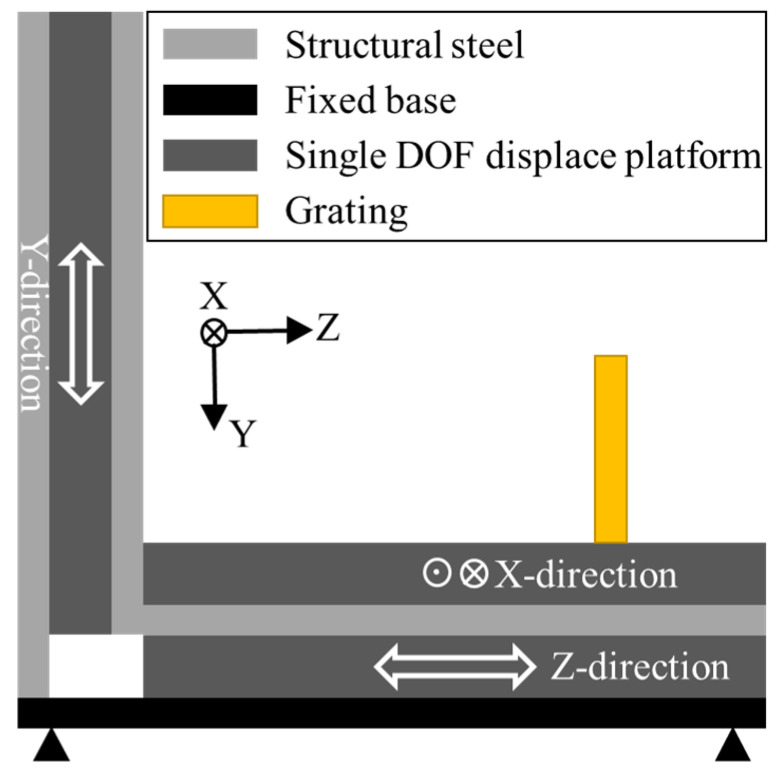
Displacement driver designed for the three-DOF reading head.

Figure 4 shows the displacement driver designed for a three-DOF reading head, which can output displacements in three directions simultaneously or one output alone. However, a cosine error is observed in the actual test. Therefore, the displacement in one-direction measurement will still output the displacement in other directions in the test and will be measured using the three-DOF encoder.

The three-DOF measurement experiment was conducted on the basis of this experimental platform. First, the pre-experiment was performed. The displacement value input by the stage controller is taken as a standard reference. Then, the following corrections were performed in accordance with the pre-experiment. The grating pitch in the X/Y direction of the grating and the wavelength of the light wave are corrected after the pre-experiment, and the corrected values are 1.1340, 1.0196, and 0.6879 μm. This paper uses a light spot with a diameter of 0.5 mm for experiments to further miniaturize the encoder. Therefore, the theoretical limit measurement range in the Z direction is 250 μm, while the range in the X and Y directions is mainly determined using the area of the measurement grating. However, the encoder will fail to perform the measurement when the displacement in the X and Y directions is excessively large and the crosstalk displacement in the Z direction exceeds the theoretical limit of 250 μm due to the cosine error. Therefore, the ranges selected for the experiment are X-250, Y-200, and Z-100 μm.

Table 2 shows the measurement results of the three DOF in the main direction of the X, Y, and Z directions. The driver velocities are 500 μm/s and 100 μm/s in the X and Y directions, respectively. The drive velocities are 1 μm/s for Z1 and Z2 and 4 μm/s for Z3. The last column of Table 2 shows the percentage error of the main displacement measurements from the input values. The table reveals the following: the minimum displacement error percentage in the X, Y, and Z directions can reach 0.0708%, 0.1389%, and 0.3339%, respectively, and the minimum errors are below 200, 300, and 400 nm, respectively. In the six groups of experiments, statistics are established on the accuracy error of the main displacement, and the average error is 455 nm. In addition to the main motion direction, measurements in the two other directions during all the tests are also stable. This finding proves the stability and accuracy of measurements in three DOF.

**Figure 5:**
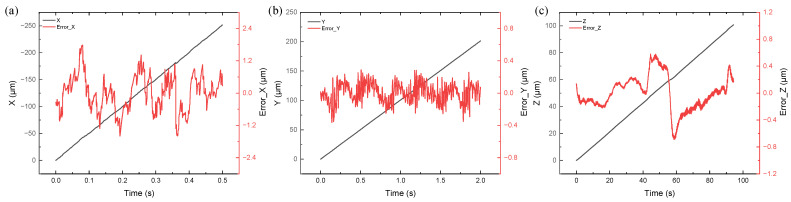
Displacement measurement results and errors of three-DOF: (**a**) X direction; (**b**) Y direction; (**c**) Z direction.

Figure 5 shows that the measurement data (plotted in black) is first linearly fitted, and the error (plotted in red) is the difference between the measured value and the fitted curve. The fitted curve equation shown below assumes that the driver velocity is constant.

X/Y/Z = V × T,(12)

where the X/Y/Z are the fit values, V is the driver velocity, and T is the time of movement.

Figure 5a shows that the predominant fluctuations in displacement measurements in the X direction are within ±2 μm mainly due to the unideal speed of the driver operation during motion. Periodic fluctuations are observed in the straight-line error of the drive screw. In addition, a problem with speed fluctuations is found in the Y and Z directions, which is considered a common problem for screw-driven drivers. The displacement velocity is the largest in all three directions, thus also demonstrating the largest displacement fluctuation. Figure 5b illustrates that the fluctuation error is less than 400 nm in the Y direction. Figure 5c shows that the fluctuation error is less than 800 nm in the Z direction. In other words, the displacement fluctuation of the PI displacement driver is larger than that of the THORLABS displacement driver. This finding is consistent with the repeatability parameter trend shown in Table 1.

An equal displacement test of 100 μm was conducted in the opposite direction of the X and Y directions to test the simultaneous measurement capability of three DOF, and the movements of the Y and X directions were turned on successively. The experimental results are X (−100.7862 μm), Y (−98.0961 μm), and Z (−1.2153 μm), and the measurement error percentages in the X and Y directions are 0.7862% and −1.9039%, respectively. The measurement error in the Y direction increases by approximately two times compared with the single movement in the Y direction due to the simultaneous movement of the two directions. A crosstalk error should be observed between the two movements of the X and Y directions.

The final test conclusion indicates that the structure can achieve submicron accuracy at 250 μm (X direction), 200 μm (Y direction), and 100 μm (Z direction), and the overall structure is small with a size of 12.3 × 7.7 × 3 cm^3^. The measurement range and accuracy can further increase with this three-DOF measurement method after the improvement of the grating processing technology in the future.

## 4. Conclusions

A compact and high-precision three-DOF grating displacement encoder is established in this paper based on the QFP prism, and the photoelectric signal is stabilized and improved through the optical path subdivision module. Three DOF can be measured simultaneously, the accuracy of the main displacement is below 500 nm on average, and the minimum error is 7.08‱. The grating processing quality and area will be further improved in the future. Therefore, the performance of the grating encoder will be further enhanced to achieve a range of millimeters and an accuracy of 1‱.

## Figures and Tables

**Table 1:** Driver brand name and performance parameter.

Instrument	Brand and Model Name	Repeatability/μm	Resolution/μm
X-displacement driver	THORLABS/Z825B	0.2000	0.0500
Y-displacement driver	THORLABS/Z825B	0.2000	0.0500
Z-displacement driver	PI/M-112	0.2500	0.0500

**Table 2:** Measurement results of three DOF in X, Y, and Z directions. The bold indicates main displacements.

Motion Axis	Input Displacement/μm	Measured Value			
		X/μm	Y/μm	Z/μm	Motion Error/%
X1	250	**−251.3361**	2.8269	−0.6948	0.5344
X2	250	**−248.8365**	5.3853	−0.5163	−0.4654
X3	250	**−249.8230**	4.4111	−0.8086	−0.0708
Y1	200	−4.0984	**200.7770**	−4.6850	0.3885
Y2	200	−2.4667	**198.9402**	−3.5440	−0.5299
Y3	200	−3.6587	**200.2778**	−5.2337	0.1389
Z1	100	−1.1824	1.1926	**102.8422**	2.8422
Z2/Reverse movement	100	0.9627	−1.0207	**−** **99.0848**	−0.9152
Z3	100	−0.0091	0.0088	**100.3339**	0.3339

## Data Availability

The data presented in this study are available on request from the Corresponding author.
